# Radiographic prediction of lunate morphology in Asians using plain radiographic and capitate-triquetrum distance analyses: reliability and compatibility with magnetic resonance arthrography (MRA) findings

**DOI:** 10.1186/s12891-019-2483-6

**Published:** 2019-03-27

**Authors:** Ji Hun Park, Tae Wook Kang, Jimi Choi, Seul Gi Kim, Kyung-Sik Ahn, Jong Woong Park

**Affiliations:** 1 Department of Orthopaedic Surgery, Korea University Guro Hospital, Korea University College of Medicine, Seoul, South Korea; 2 Department of Orthopaedic Surgery, Korea University Anam Hospital, Korea University College of Medicine, 73, Inchon-ro, Sungbuk-gu, Seoul, 06334 South Korea; 3 Department of Biostatistics, Korea University College of Medicine, Seoul, South Korea; 4 Department of Radiology, Korea University Anam Hospital, Korea University College of Medicine, Seoul, South Korea

**Keywords:** Lunate type, Medial hamate facet, MR arthrography, Lunate morphology

## Abstract

**Background:**

The purpose of this study was to examine the reliability of plain radiographic methods of determining the lunate type and its compatibility with magnetic resonance arthrography (MRA) findings.

**Methods:**

Plain radiographs of a total of 150 wrists were reviewed by three observers. Lunate types were evaluated using both conventional posteroanterior (PA) radiographic analysis and the capitate-triquetrum distance (CTD) analysis. Cohen kappa and Fleiss kappa statistics were used to estimate intra- and inter-observer reliabilities. Compatibility with the MRA findings, as assessed by each observer, was investigated.

**Results:**

The overall intra-observer reliability was 0.517 for the analysis and 0.589 for the CTD analysis. The overall inter-observer agreement was 0.448 for the PA radiographic analysis and 0.581 for the CTD analysis. The PA radiographic analysis and MRA findings for the detection of medial lunate facets were compatible in 119 of the 150 patients (79.3%). Twenty-eight (90.3%) of the 31 incompatible wrists had a medial facet on MRA (Type II), which was not detected in the PA radiographic analysis. In the CTD analysis, the results for 27 of 29 Type II lunates (93.1%) and 39 of 45 Type I lunates (86.7%) were compatible with the MRA.

**Conclusions:**

This study suggests that predicting the lunate type by plain radiographs alone is insufficient, as both radiographic analyses showed moderate intra- and inter-observer reliabilities. Although both radiographic analyses showed good compatibility with the MRA for Type II lunates, clinicians should be alert to undetected medial facets in Type I lunates on PA radiographic analysis.

## Background

As a keystone of the wrist, the lunate is integrated into the central column and proximal row of the carpus. Substantial axial and torsional loads from the carpal bones and surrounding carpal ligaments are often concentrated on the lunate [1, 2]. The clinical effects of morphologic variances in the lunate are unknown, but there is concern that they could play a role in various patterns of carpal instability.

Two major types of the lunate have been described according to the absence (Type I) or presence (Type II) of a medial facet distally articulating with the hamate [3]. Biomechanical and motion analysis studies have revealed variations in lunate morphology, and associated adaptations to the intercarpal ligaments could influence carpal kinematics and predispose them to asymmetric loading [4–7]. To facilitate use in a clinical setting, researchers have translated the classifications devised from a cadaveric study into posteroanterior (PA) radiographic analysis; previously, this method has been used to determine the lunate types [8].

Nevertheless, a medial facet of the lunate is not always distinguishable on PA radiographs [8]. A study on radiographic prediction of the lunate showed that wrist PA radiography had 64 to 72% accuracy [9]. To minimize the possibility of including some Type II lunates with a small medial facet in the Type I group, Nakamura et al. [6] used the shortest distance between the capitate and triquetrum on a posteroanterior radiograph to identify Type I and Type II lunates. However, fair agreement between the lunate types from capitate-triquetrum distance (CTD) analysis and PA radiographic analysis has been reported, and only a few studies have assessed the reliability of these methods [10, 11].

Bone morphologic evaluation has also involved various imaging modalities, such as computed tomography and magnetic resonance imaging (MRI) [5, 7]. With the recent development of better spatial resolution and the use of microscopy coils, MRI shows good performance in visualizing the small joint anatomy [12, 13]. Magnetic resonance arthrography (MRA) has even higher accuracy than conventional MRI in detecting wrist joint pathology [14, 15]. Several MRI and MRA studies have demonstrated well-correlated morphologic evaluations of the lunate with cadaver dissection, as MRI has the ability to image the geometric properties of this small bone, as well as the status of the articular cartilages [16, 17].

The purpose of this study was to examine the reliability of the two current methods for determining the lunate type: PA radiographic analysis and CTD analysis. Further, we sought to evaluate the compatibility of the radiographic classification of lunate types with MRA findings.

## Methods

Institutional review board approval was obtained for this study. A single-center retrospective analysis was performed by using medical records and imaging studies of patients who underwent both a plain radiographic series and MRA of the wrist at our institution between 2015 and 2017. Pfirrnann et al. [17] showed that MRA produces lunate facet findings that are consistent with those from cadaveric dissection; thus, MRA was evaluated for comparison with plain radiographic findings in the current study. Patients older than 18 years but younger than 65 years and those with optimal-quality MRA images showing the lunate facets were included. Patients with inadequate wrist position, carpal instability, or carpal arthritis on radiographs and abnormal lunate changes on imaging analysis, such as avascular necrosis, were excluded [10].

A total of 150 patients (86 men and 64 women) were evaluated and radiographs taken from one of their wrist (89 right and 61 left) were used in this study. The patients ranged in age from 19 to 56 years (mean, 36 years). All patients were of Asian descent and had undergone a wrist radiographic series according to the protocol at our institution, comprising neutral, maximal radial deviation, maximal ulnar deviation, and pronated grip PA and lateral views. All patients also underwent MRA of the wrist.

### Radiographic prediction of the lunate type

**Fig. 1:**
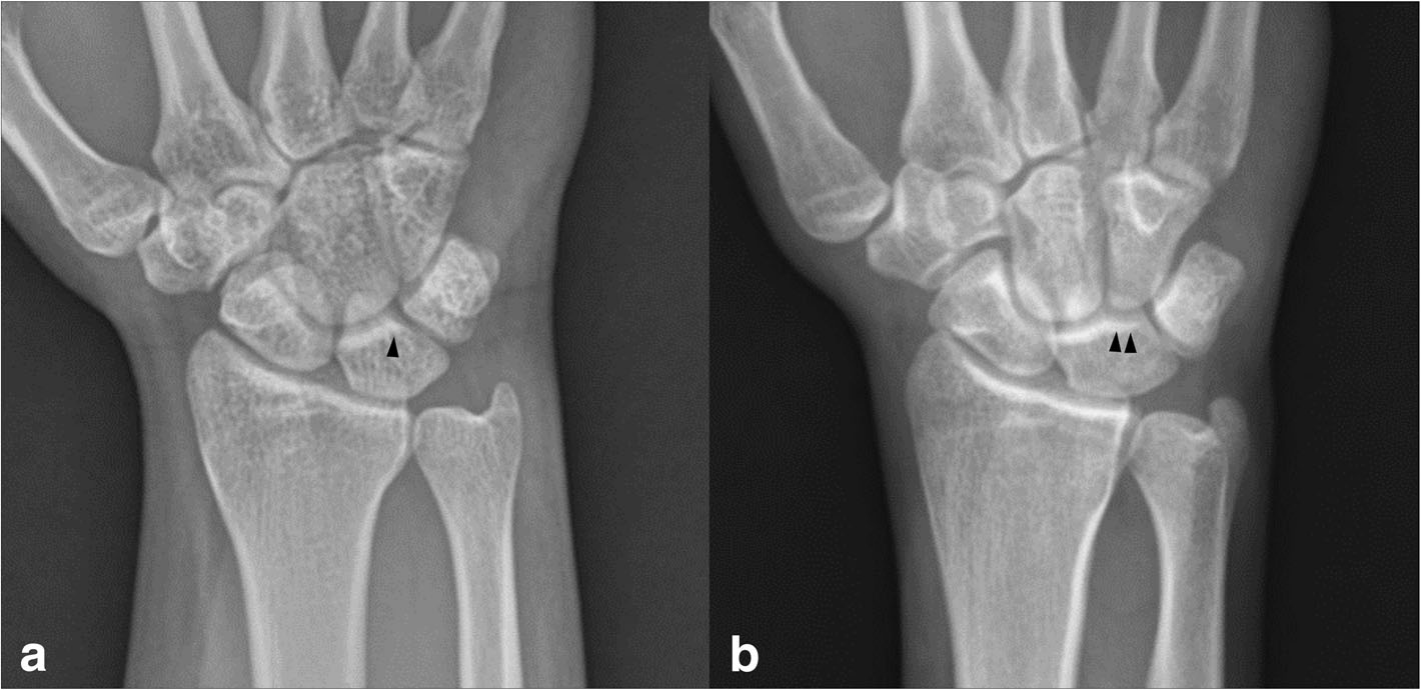
Examples of (**a**) Type I and (**b**) Type II lunates classified by plain radiographic analysis; arrowheads point to the absence or presence of a medial hamate facet on the lunate, respectively

**Fig. 2:**
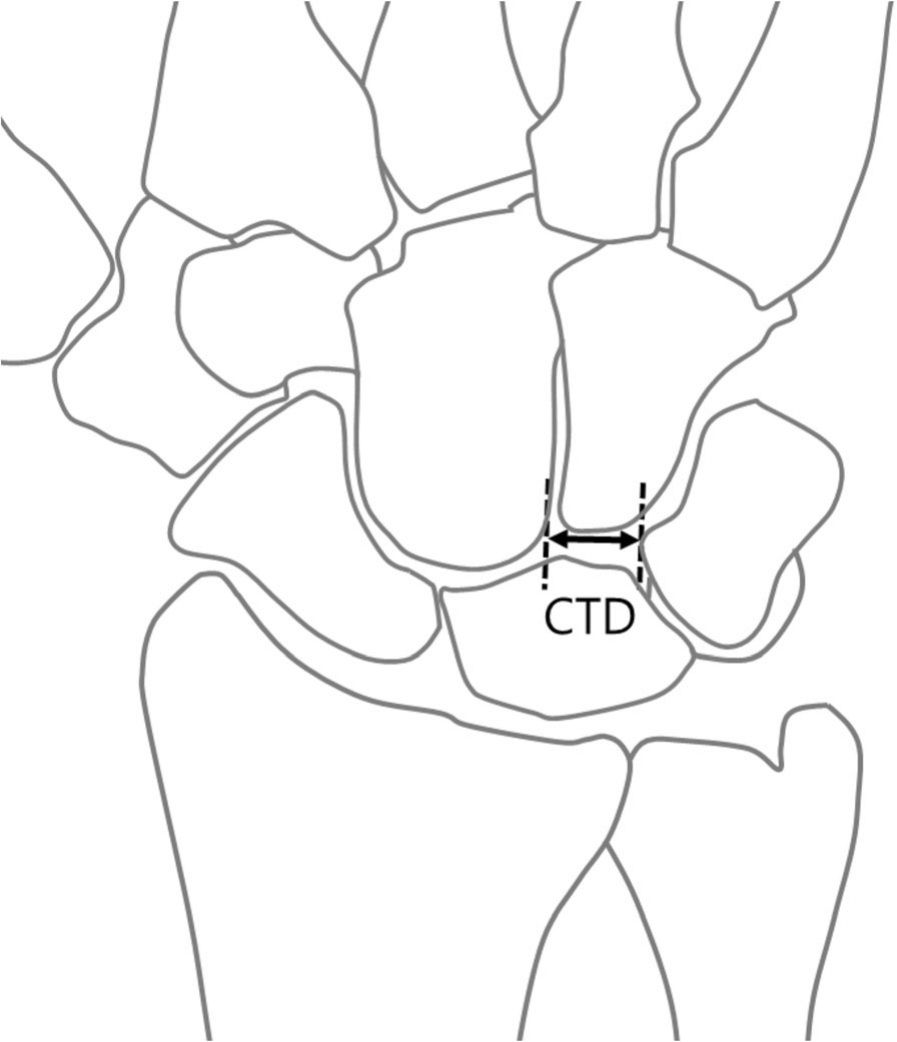
Measurements of capitate-triquetrum distance (CTD). CTD is defined as the shortest distance between the capitate and triquetrum

Two radiographic analysis methods were used to determine the lunate type on plain radiographs. First, the classification of Viegas et al. [8] was used: the lunate type was classified as Type I or II via radiographic detection of a medial facet of the lunate (PA radiographic analysis) (Fig. 1). If a medial facet that did not line up with the entire distal articular line of the lunate was detected on at least one posteroanterior wrist radiograph among those taken in various positions (neutral, maximal radial deviation, maximal ulnar deviation, and pronated grip view), it was considered a Type II lunate. Second, the CTD analysis suggested by Galley et al. [11] was used (Fig. 2) In this method, the lunate type was classified on the basis of the CTD, which was defined as the shortest distance between the capitate and triquetrum on a neutral PA radiograph. A Type I lunate had a CTD of ≤2 mm, while a Type II lunate had a CTD ≥ 4 mm; an intermediate group lay between these values.

Three observers (two board certified orthopedic surgeons and one senior resident) who were educated on radiographic analysis independently reviewed each radiograph to determine the lunate type. Each observer repeated the analysis for the second time at least 2 weeks after the previous review. The observers were blinded to their previous findings. Each observer measured the CTD of randomly assigned wrist radiographs and were blinded to the lunate types from the previous PA radiographic analysis. All measurements were performed by using digital angle and distance-measuring tools integrated to a picture archiving and communication system.

### Magnetic resonance arthrography

**Fig. 3:**
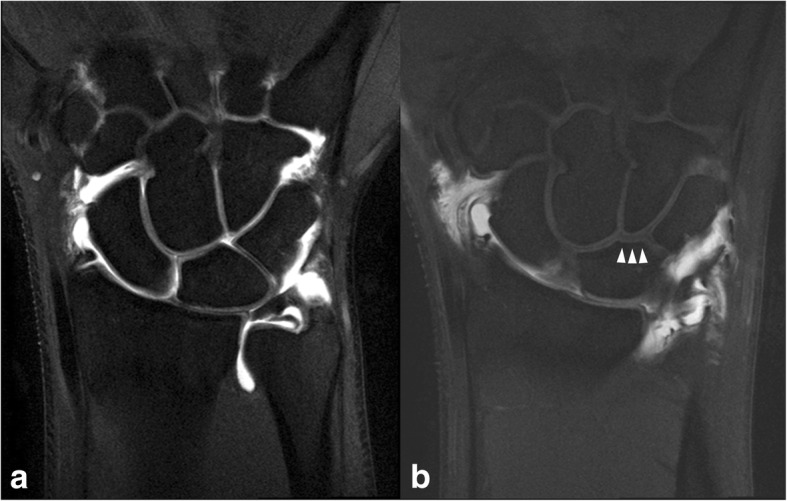
Examples of the detection of a medial hamate facet of the lunate on magnetic resonance arthrography. Coronal fat-saturated, turbo spin-echo, T1-weighted arthrographic images of (**a**) Type I lunate and (**b**) Type II lunate with a medial hamate facet (arrowhead)

MRA was performed with a 3.0-T magnetic resonance whole-body scanner (Achieva, Philips Medical Systems, Best, The Netherlands) using a dedicated eight-channel array wrist coil (SENSE wrist coil 8, Philips Medical Systems) within 30 min after injection of a contrast agent. The wrist was placed in the prone position parallel to the long axis of the gantry with the center of the bore determined by a laser mark. The sequences examined were fat-saturated T1-weighted and T2-weighted turbo spin-echo sequences in the coronal plane (slice thickness, 2.00 mm) and fat-saturated THRIVE turbo fluid echo sequences in the coronal plane (slice thickness, 0.45 mm). The images were viewed with picture archiving and communication system. One board certified orthopedic surgeon who specialized in hand surgery and one musculoskeletal radiologist independently analyzed lunate morphology on the MRA scans and classified the lunates as Type I or II. In cases of disagreement, the final type was decided by consensus. A medial facet of the lunate was defined as an additional midcarpal joint surface at the distal surface of the lunate bone that was clearly distinguishable from the main distal joint surface with either a concave or straight surface and was visible on at least two continuous slices, as suggested by Pfirrmann et al. [17] (Fig. 3). A convex surface was not considered to be a distinct facet [16].

### Statistical analysis

Intra-observer reproducibility was assessed by comparing the observations made by the same observer during the first and second evaluations using the Cohen kappa coefficient. Inter-observer reliability was assessed by comparing the observations made by each observer during the first evaluation using the Fleiss kappa coefficient to accommodate multiple raters. The 95% confidence interval for the kappa value was estimated. Kappa values were interpreted according to the Landis and Koch criteria, where a kappa value of > 0.80 suggests almost perfect agreement, 0.61–0.80 suggests substantial agreement, 0.41–0.60 suggests moderate agreement, 0.21–0.40 suggests fair agreement, 0–0.20 suggests slight agreement, and a negative kappa value suggests poor agreement [18]. Compatibility between the PA radiographic analysis findings of each observer and the MRA findings was assessed to determine the accuracy of radiographic prediction. Statistical analyses were conducted with R (version 3.3.2) using the psych and raters packages. A *p*-value of < 0.05 was considered statistically significant.

## Results

The overall multi-rater generalized Fleiss kappa value for inter-observer agreement of the three raters was 0.448 for the PA radiographic analysis (moderate agreement) and 0.581 for the CTD analysis (moderate agreement) (Table 1).

**Table 1:** Inter-observer agreement among the three raters

	Assessment	Fleiss kappa	95% CI of kappa	Interpretation
PA radiographic analysis	1st reading	0.515	0.421, 0.606	Moderate
	2nd reading	0.383	0.541, 0.674	Fair
	Overall	0.448		Moderate
CTD analysis	1st reading	0.608	0.541, 0.674	Moderate
	2nd reading	0.554	0.487, 0.620	Moderate
	Overall	0.581		Moderate

*CI* confidence interval, *PA* posteroanterior, *CTD* capitate-triquetrum distance

The Cohen kappa value for intra-observer reproducibility of the PA radiographic analysis was the highest for Observer 1 (0.570) and lowest for Observer 2 (0.446); the overall kappa value was 0.517 (moderate agreement). The linearly weighted kappa value for intra-observer reproducibility of the CTD analysis was highest for Observer 1 (0.660) and lowest for Observer 2 (0.540); the overall kappa value was 0.589 (moderate agreement) (Table 2).

**Table 2:** Intra-observer agreement among the three raters

	Assessment	Cohen kappa	95% CI of kappa	Interpretation
Observer 1	PA radiographic analysis	0.570	0.416, 0.724	Moderate
	CTD analysis	0.660^a^	0.528, 0.792	Substantial
Observer 2	PA radiographic analysis	0.446	0.282, 0.610	Moderate
	CTD analysis	0.540^a^	0.383, 0.688	Moderate
Observer 3	PA radiographic analysis	0.552	0.409, 0.696	Moderate
	CTD analysis	0.567^a^	0.411, 0.722	Moderate
Overall	PA radiographic analysis	0.517		Moderate
	CTD analysis	0.589^*^		Moderate

^a^weighted Kappa

*CI* confidence interval, *PA* posteroanterior, *CTD* capitate-triquetrum distance

Among the 150 wrists evaluated via MRA, 84 (56.7%) were Type I lunates, and 66 (42.7%) were Type II. The PA radiographic analysis and MRA findings for the detection of medial facets of the lunate were compatible in 119 of the 150 patients and incompatible in 31 patients. The overall compatibility between the PA radiographic analysis and MRA findings for lunate morphology was 79.3% (Table 3).

**Table 3:** Comparison of the PA radiographic analysis and MRA findings

	Type II lunate (MRA findings)	Type I lunate (MRA findings)	Total
Type II lunate (PA radiographic analysis)	38 (25.3%)	3 (2%)	41 (28%)
Type I lunate (PA radiographic analysis)	28 (18.7%)	81 (54.7%)	109 (73.3%)
Total	66 (42.7%)	84 (56.7%)	150 (100%)

Compatibility of Type II lunate = 38/41 = 92.7%. Compatibility of Type I lunate = 81/109 = 74.3%. *PA* posteroanterior, *MRA* magnetic resonance arthrography

**Fig. 4:**
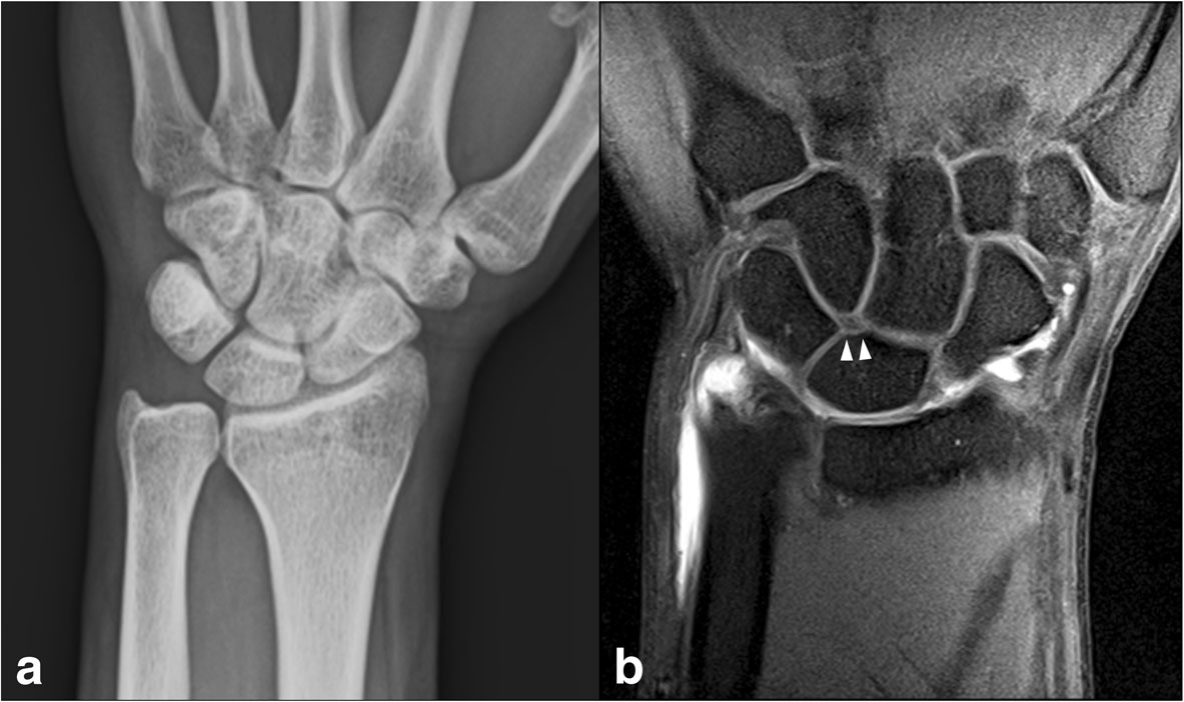
A 37-year-old man with wrist pain. (**a**) Wrist plain radiograph recorded as Type I lunate on plain radiographic analysis. (**b**) Coronal fat-saturated T1-weighted arthrographic image showing a radiographically undetected medial facet of the lunate (arrowhead)

Twenty-eight (90.3%) of the 31 incompatible wrists had a medial facet of the lunate on MR arthrography, which was not detected in the PA radiographic analysis (Fig. 4). Three (9.7%) of the incompatible wrists showed no evidence of a medial facet on MRA, but were classified as Type II lunates in the PA radiographic analysis.

In the CTD analysis, 76 (50.7%) of wrists were classified into the intermediate group, which showed a similar distribution of Type II lunates (22%) and Type I lunates (28.7%) compared with MRA findings. When the wrists of the intermediate group were excluded from the analysis, 27 of the 29 Type II lunates (93.1%) and 39 of the 45 Type I lunates (86.7%) on the CTD analysis were compatible with the MRA findings (Table 4).

**Table 4:** Comparison of CTD analysis and MRA findings

	Type II lunate (MRA findings)	Type I lunate (MRA findings)	Total
Type II lunate (CTD analysis)	27 (18%)	2 (1.3%)	29 (19.3%)
Intermediate lunate (CTD analysis)	33 (22%)	43 (28.7%)	76 (50.7%)
Type I lunate (CTD analysis)	6 (4%)	39 (26%)	45 (30%)
Total	66 (44%)	84 (56%)	150 (100%)

Compatibility of Type II lunate = 27/29 = 93.1%, Compatibility of Type I lunate = 39/45 = 86.7%, *CTD* capitate-triquetrum distance, *MRA* magnetic resonance arthrography

## Discussion

The clinical significance of different lunate types has recently been highlighted. Lunate morphology can be classified by the absence (Type I) or presence (Type II) of a medial facet that contacts the proximal pole of the hamate [3]. Several studies have reported that this additional articulation can alter load transmission across the radiocarpal joint and exert significant influence on carpal kinematics [4, 6, 7] A protective effect against carpal instability associated with scaphoid nonunions and scapholunate dissociation in Type II lunate has been reported [10]. Similarly, Rhee et al. [5] reported that Type II lunate can inhibit abnormal scaphoid flexion deformity in Kienböck disease and may slow the progression of lunate fracture and fragmentation. Lunate classification according to the medial facet has used simple radiographs, but few studies of the reliability of this method are available.

Plain radiographic prediction of lunate morphology has some limitations [9]. Several studies have suggested radiographic characteristics of adjacent carpal bone shapes and alignment to determine the lunate type; however, their interpretation still depends on eidetic impressions of the presence of a medial facet on the lunate. Further, a small difference in the CTD might be the result of inherent bone size differences or measurement errors. The current study assessed intra-observer reliability and inter-observer agreement of two commonly used classification systems for radiographic lunate types and their compatibility with MRA images. Both systems had moderate intra- and inter-observer reliabilities; nevertheless, the CTD analysis showed more consistent agreement than did the PA radiographic analysis. The estimated Kappa value for inter-observer reliability was 0.554 with 95% confidence interval of 0.487 to 0.620. This suggests that the true value of Kappa is higher than 0.487 with 95% confidence. The overall compatibility with MRA for plain radiographic prediction was ~ 79.3%. When the lunates were interpreted to have a medial facet (Type II) on radiographic analyses, they showed good compatibility with the MRA findings. However, when the lunates were interpreted as Type I, there were more cases of mismatch, in which undetected medial facets were observed on MRA.

A possible explanation for the discrepancy in the lunate type on plain radiographs, especially among observers, is that variable size and geographic differences in the medial facet of the lunate may be confusing to some observers. Specifically, the observers had difficulty in interpreting a smaller medial facet, which sometimes overlapped with a lateral facet for capitate articulation and the ulnar margin of the lunate on the PA view. Although the anatomical diversity of the medial facet of the lunate is not fully understood, we observed several discrepancies among the observers when the angle of the medial facet with the lateral facet was less acute. To add to this difficulty, the medial facet is often larger dorsally and may be completely absent on the most volar side. A cadaveric study showed 77% occupation of the lunate medial facet. Pfirmann et al. [17] reported that the size of the medial facet is not uniform throughout consecutive coronal images on MRA. Some of the medial facets did not occupy the entire anteroposterior width of the lunate and were absent in the most volar coronal images. When the lunate occupied a lesser anteroposterior width, the radiographic articular line may not have clearly reflected the presence of a medial facet. Thus, although the lunate types looked clear pictographically, the types were sometimes interpreted subjectively, leading to variability in the inter- and intra-observer reliabilities.

The incidence of Type II lunates seems to vary in different populations [5]. The rate has been reported to range from 27 to 73% worldwide. In the largest study of an Asian population, Tatebe et al. [19] reported a 45% incidence rate in 637 cases of midcarpal arthroscopy. Arai et al. [20] reported a 57.5% incidence of Type II lunates in 127 cadaveric wrists. The incidence of Type II lunates in our study was 42.7%, which is similar to that of previous reports. Most incompatible cases were Type I lunates determined via PA radiographic analysis; in contrast, Type II lunates showed an almost perfect match between the PA radiographic analysis and MRA findings. This study showed a 92.7% compatibility rate of Type II lunates with the MRA findings; Type I lunates showed a lower compatibility rate of 74.3%. These findings suggest that clinicians should be alert to undetected medial facets of the lunate in patients with Type I lunate determined by PA radiographic analysis. In the CTD analysis, Types I and II both showed good compatibility with the MRA findings, and the evaluations by the same observer were more consistent. Both Type I lunate (CTD ≤ 2 mm) and Type II lunate (CTD ≥ 4 mm) showed a strong match with the MRA findings. However, we could not determine the superiority of the CTD analysis to PA analysis. More than half of the patients were classified into the intermediate group in the CTD analysis, and this group showed a similar distribution of Type I and Type II lunates on MRA. The clinical effects of an intermediate lunate type are unknown; thus, further studies are warranted to examine the effects on wrist pathology or kinematic characteristics of the intermediate type.

Several limitations of this study need to be acknowledged. Although we used static images, which did not allow simulation of carpal motions, the actual articulation of detectable facets was not verified. Instead, the articulating facets were predicted from MRA, which provided serial slices of the thinly sectioned articular surface and cartilage shape. Although the original classification of lunate type, derived from a cadaveric dissection, included a medial lunate facet width of less than 1 mm [3], we used a stricter definition for a medial hamate facet in accordance with a previous MRA study [17]. There is still controversy as to whether lunates with a very small facet have clinical significance. Although it is possible that we underestimated the number of Type II lunates, it should be noted that the proportion of Type II lunates evaluated using MRA in our cohort was similar to that of previous studies of the same ethnicity [19, 20].

## Conclusion

This study suggests that predicting the lunate type by plain radiographs alone is insufficient. Both radiographic analyses showed moderate intra- and inter-observer reliabilities. Although the Type II lunates showed good compatibility between the radiographic analyses and MRA findings, clinicians should be alert to undetected medial facets in Type I lunates on PA radiographic analysis.

## Abbreviations

CI: Confidence interval
CTD: Capitate-triquetrum distance
MRA: Magnetic resonance arthrography
MRI: Magnetic resonance imaging
PA: Posteroanterior
THRIVE: High resolution isotropic volume excitation
